# Supplementary material for: Tumor Promoting Effects of Sulforaphane on Diethylnitrosamine-Induced Murine Hepatocarcinogenesis
Source: Int J Mol Sci. 2022 May 12;23(10):5397. doi: 10.3390/ijms23105397 (PMC9141104; doi:10.3390/ijms23105397)
Supplement: Supplementary file 1 [file ijms-23-05397-s001.zip › ijms-1676433-supplementary.pdf]

Supplementary Table S1. Detailed information of all liver specimens from vehicle- and DEN-treated mice.

Specimen ID	Genotype	Base Position	Genotype Result	User Edit	QV	Base Position Coverage	Amplicon ID	Layer ID	ROI ID	ROI Position	Genotype Comments
DEN1-1004	1910t>W	1910	-	no	65	2X	Mus_Braf_V637E	NP_647455.3	NP_647455.3_region 1	2077	
DEN2-1004	1910t>W	1910	-	no	65	2X	Mus_Braf_V637E	NP_647455.3	NP_647455.3_region_1	2077	
DEN3-1004	[=]+[=]	1910	-	no	65	2X	Mus_Braf_V637E	NP_647455.3	NP_647455.3_region 1	2077	
DEN4-1004	1910t>W	1910	-	no	52	2X	Mus_Braf_V637E	NP_647455.3	NP_647455.3_region_1	2077	
DEN5-1004	[=]+[=]	1910	-	no	65	2X	Mus_Braf_V637E	NP_647455.3	NP_647455.3_region 1	2077	
DEN6-1004	1910t>W	1910	-	no	65	2X	Mus_Braf_V637E	NP_647455.3	NP_647455.3_region_1	2077	
DEN7-1004	[=]+[=]	1910	-	no	65	2X	Mus_Braf_V637E	NP_647455.3	NP_647455.3_region 1	2077	
NC1-1004	[=]+[=]	1910	-	no	49	2X	Mus_Braf_V637E	NP_647455.3	NP_647455.3_region_1	2077	
NC2-1004	[=]+[=]	1910	-	no	65	2X	Mus_Braf_V637E	NP_647455.3	NP_647455.3_region 1	2077	
NC3-1004	[=]+[=]	1910	-	no	47	2X	Mus_Braf_V637E	NP_647455.3	NP_647455.3_region_1	2077	
NC4-1004	[=]+[=]	1910	-	no	65	2X	Mus_Braf_V637E	NP_647455.3	NP_647455.3_region 1	2077	
NC5-1004	[=]+[=]	1910	-	no	65	2X	Mus_Braf_V637E	NP_647455.3	NP_647455.3_region_1	2077	
NC6-1004	[=]+[=]	1910	-	no	46	2X	Mus_Braf_V637E	NP_647455.3	NP_647455.3_region 1	2077	
NC7-1004	[=]+[=]	1910	-	no	40	2X	Mus_Braf_V637E	NP_647455.3	NP_647455.3_region_1	2077	

A

Primer name	Sequence	PCR size (bp)	Tm.
Mus_Braf_V637E_2F_M13F	GTAAAACGACGGCCAGTTTCCTTTACTTACTGCAC	290	60
Mus_Braf_V637E_R	GCAATTATGCCTGGCTTACA		

B

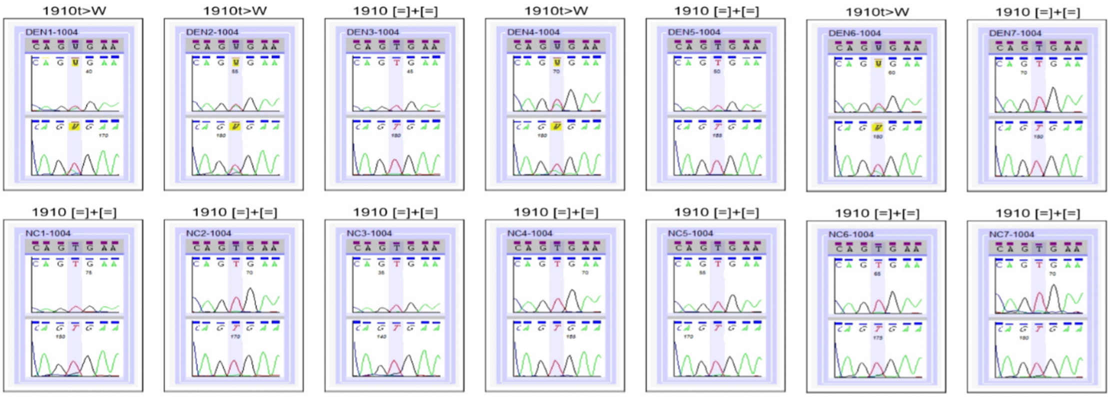

Supplementary Figure S1. ***B-Raf*** ^{V637E} **mutation detected in DEN-induced liver tumors.** Total RNA was extracted from vehicle- or DEN-treated liver tissue with the DNeasy® Blood & Tissue Kit, and converted to cDNA using reverse transcriptase

following the standard procedure. The cDNA region bearing murine *B-Raf* gene was amplified using PCR with HiPi Tag polymerase. The PCR products were purified by the QIAquick Purification Kit. **(A)** Designed primers for sequencing are listed, GTAAAACGACGGCCAGT sequence represents M13F primer. **(B)** Sequences of the genetic variants in murine *B-Raf* ^{V637E} were detected by capillary electrophoresis.

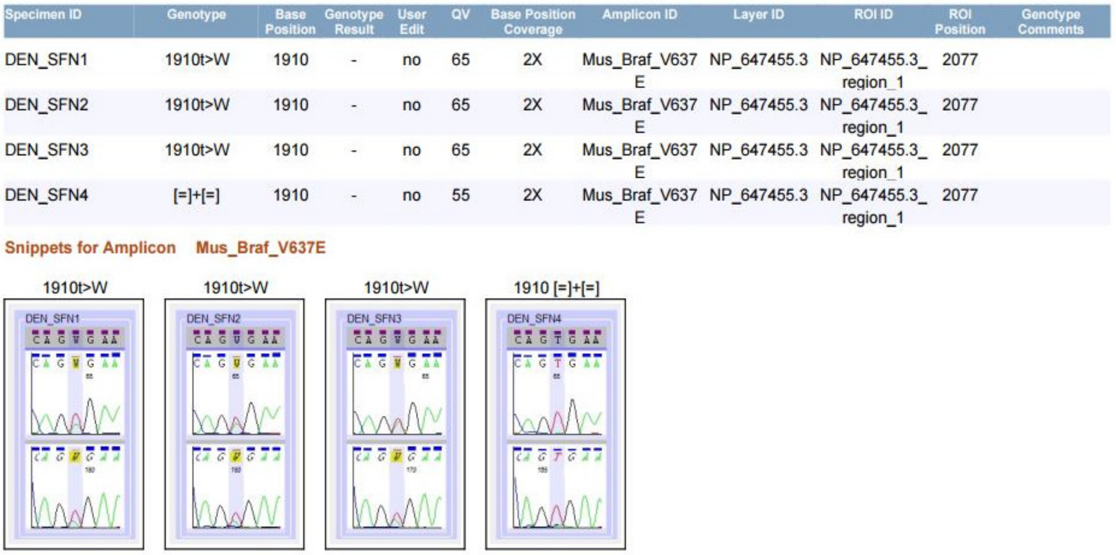

Specimen ID	Genotype	Base Position	Genotype Result	User Edit	QV	Base Position Coverage	Amplicon ID	Layer ID	ROI ID	ROI Position	Genotype Comments
DEN_SFN1	1910t>W	1910	-	no	65	2X	Mus_Braf_V637E	NP_647455.3	NP_647455.3_region_1	2077	
DEN_SFN2	1910t>W	1910	-	no	65	2X	Mus_Braf_V637E	NP_647455.3	NP_647455.3_region_1	2077	
DEN_SFN3	1910t>W	1910	-	no	65	2X	Mus_Braf_V637E	NP_647455.3	NP_647455.3_region_1	2077	
DEN_SFN4	[=]+[=]	1910	-	no	55	2X	Mus_Braf_V637E	NP_647455.3	NP_647455.3_region_1	2077	

Supplementary Figure S2. *B-Raf* ^{V637E} mutation detected in SFN-treated DEN-induced liver tumors. Sequencing was performed and cleaned up with the BigDye® Terminator v3.1 Cycle Sequencing Kit. Sequences were detected and analyzed by ABI PRISM 3730XL Analyzer. Sequences of the genetic variants in murine *B-Raf* ^{V637E} were detected by capillary electrophoresis. Three out of 4 cases of *B-Raf* ^{V637E} mutation (labelled by yellow color) were detected by capillary electrophoresis in SFN plus DEN-induced liver tumors.

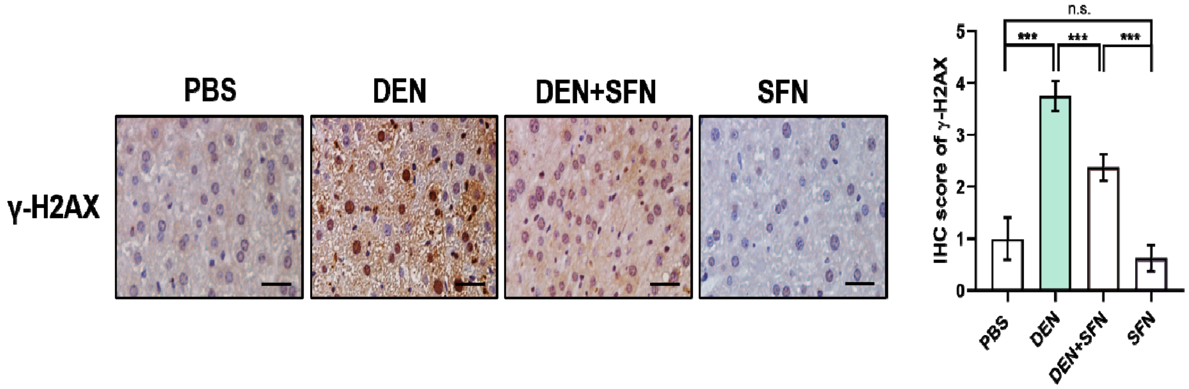

Supplementary Figure S3. Immunohistochemical analysis of γ-H2AX in DEN-induced murine hepatocarcinogenesis with and without SFN administration. The paraffin sections of liver tissues were subjected to immunohistochemical staining with an antibody against γ-H2AX. The IHC score was analyzed by the image processing program Image ,J and results are shown as the mean ± SD of 4 samples for each group. ***$p < 0.001$; n.s.: non-significant. Representative images of stained sections are displayed. Scale bar, 100 μm.
